# Dr. Shapter, on a Case of Delirium

**Published:** 1800-03

**Authors:** W. R. Shapter

**Affiliations:** Inspector of Hospitals


### *To the Editors of the Medical and Physical Journal.*

*Harwich, General Hospital,*

*January* 27, 1800.

Gentlemen,

If the following Case, to which I was called in, a few hours previous to its fatal termination, should appear to you as it does to me, remarkable in some of its circumstances, I beg you will insert it in your very useful publication; and am,

Gentlemen,

Your obedient humble servant,

W. R. SHAPTER, M.D.

Inspector of Hospitals.

M. B. aged 22, wife of a sailor belonging to a gun-vessel, being advanced in pregnancy from six to seven months, went on board with her husband on the 25th of December, 1799, and came ashore two or three days after. Her looks were remarked by her friends to be particularly wild; but she made no complaint, following her usual occupation of washing, until the evening of the 30th, when she complained of a severe head-ach. The next morning she came down stairs in a state of delirium, talking incoherently and unintelligibly, and vomited several times a bilious matter. Apprehending the approach of labour, Mr. Hopkins, surgeon of this town, was was fent for, who, on an examination, finding no fymptoms of that kind, directed two grains of antimonial tartar to be given immediately; foon after which the vomiting ceafed. In the afternoon the delirium ftill continued, and early in the evening fhe was violently and univerfally convulfed, fucceeded by ftupor, which continued alternately through the night. Her teeth were clofed, fo that no liquid could be got down, and fhe frothed very much at the mouth. Pulfe fmall and quick. No ftool.

On the firft of January, 1800, there were intervals of nearly an hour between each fit of convulfion, but only a fmall quantity of fluid could be adminiftered. Blifters were applied to the back and legs; and an enema was injected, which produced one moderate evacuation. She became more eafy towards evening, feeming to exprefs a forenefs of her throat and tongue; and in the middle of the night, for the firft time fpoke, faying, fhe was hungry, and eat fome mouthfuls greedily, yet did not appear to know any perfon about her.

The morning of the 2d, her fits recurred, although with lefs violence; after which fhe remained comatofe. Her head being fhaved, the fcalp appeared generally but flightly oedematous; no particular mark of injury, or fign of uneafinefs on preffure, could be difcovered. The pupil contracted freely on expofure to light. Pulfe 144. In the afternoon, a cathartic mixture was ordered; only a fmall quantity was fwallowed, which produced no effect; and a blifter was applied to her head.

3d January. She died about four o'clock this morning.

On examination after death, there was no appearance of fracture of the cranium. The membranes were redder, and the larger blood veffels more diftended than natural, but no extravafation, nor any thing uncommon in the fubftance or cavities of the brain.

In the abdomen; the uterus, of confiderable fize, contained a female foetus, which, from its putrid ftate and flabby confiftence, appeared to have been dead for a much longer time than the period of the fymptoms above recounted. The reft of the abdominal vifcera were perfectly found, except the ftomach, in which was a remarkable degree of inflammation of the internal coat, extending from the cardia in every direction about two inches, perfectly circumfcribed, and of a deep fcarlet colour, interfperfed with very minute black fpots.

The thorax was not examined.

Induced by thefe appearances to make farther inquiry of her friends, they could give no account of what had happened to to her on board the veſſel, as her huſband had ſailed; ſhe had drank half a glaſs of rum previous to her going into the boat, the crew of which were all drunk, as ſhe expreſſed on her return, and that there was ſo much confuſion among them, ſhe knew not how ſhe got on board.

She bore a character of being at all times very temperate, and not in the leaſt addicted to drinking ſpirituous liquors.

